# Type D personality, stress, coping and performance on a novel sport task

**DOI:** 10.1371/journal.pone.0196692

**Published:** 2018-04-26

**Authors:** Erika Borkoles, Mariana Kaiseler, Andrew Evans, Chantal F. Ski, David R. Thompson, Remco C. J. Polman

**Affiliations:** 1 School of Public Health and Social Work, Queensland University of Technology, Brisbane, QLD, Australia; 2 Institute for Sport, Physical Activity and Leisure, Leeds Beckett University, Leeds, Yorkshire, United Kingdom; 3 Directorate of Sport, Exercise, & Physiotherapy, The University of Salford, Lancashire, United Kingdom; 4 School of Nursing and Midwifery, Queen’s University, Belfast, Northern Ireland; 5 School of Exercise & Nutrition Sciences, Queensland University of Technology, Brisbane, QLD, Australia; Queen Mary University of London, UNITED KINGDOM

## Abstract

We investigated (1) the relationship between Type D personality, stress intensity appraisal of a self-selected stressor, coping, and perceived coping effectiveness and (2) the relationship between Type D personality and performance. In study one, 482 athletes completed the Type D personality questionnaire (DS14), stress thermometer and MCOPE in relation to a recently experienced sport stressor. Type D was associated with increased levels of perceived stress and selection of coping strategies (more emotion and avoidance coping) as well as perceptions of their effectiveness. In study two, 32 participants completed a rugby league circuit task and were assessed on pre-performance anxiety, post-performance affect and coping. Type D was associated with poorer performance (reduced distance; more errors), decreases in pre-performance self-confidence and more use of maladaptive resignation/withdrawal coping. Findings suggest that Type D is associated with maladaptive coping and reduced performance. Type D individuals would benefit from interventions related to mood modification or enhancing interpersonal functioning.

## Introduction

Competitive sport is associated with stressful experiences [1]. For athletes to perform to the best of their ability and to feel satisfied with their performance, it is essential that they use adaptive coping strategies to deal with these stressors [2, 3]. A number of factors have been found to influence the stress and coping process including the personality of athletes [4, 5]. A personality construct which has been shown to have significant consequences for behavior, health, stress, and coping but which has not been examined in the domain of sport is the distressed or Type D personality [6]. To address this research gap, two empirical studies were conducted using a crossectional and quasi-experimental design respectively, to investigate how Type-D personality in a sport context might influence (i) stress, coping preferences and perception of coping effectiveness; (ii) performance on a novel sport task. Type D personality is likely to result in maladaptive coping with stress experienced in sport thereby influencing athletic performance adversely. However, this issue has not been examined to date. As such this research might have important implications for athletes and practitioners.

The cognitive-motivational-relational model of stress and coping describes the relation between stress and coping as a dynamic process between an individual’s internal and situational environment [7, 8]. Through primary appraisal an individual will assess the significance of an event to their personal values, beliefs or intentions. Secondary appraisal involves the complex evaluative process in which a person analyses their available coping options in relation to the specific situation, maximizing gains or favorable outcomes and limiting harm [8]. Perceived stress intensity [9] as well as perceptions of control [10] have been found to be factors influencing the coping process.

Coping has been defined as “a constantly changing cognitive and behavioral effort to manage specific external and/or internal demands that are appraised as taxing or exceeding the resources of the person” (p.141) [8]. Coping responses have been classified into higher order dimensions. Nicholls and Polman [11] in their systematic review on coping in sport suggested three dimensions. First, problem-focused coping refers to cognitive and behavioral attempts to manage distress by reducing or eliminating the stressor. Second, emotion-focused coping is concerned with the regulation of emotional arousal and distress [7]. Finally, avoidance coping includes behavioral and psychological efforts to disengage from a stressor [12]. Endler and Parker [13], on the other hand, made a distinction between approach- (efforts to deal with a stressful situation) and avoidance-oriented (cognitive changes to avoid confrontation) coping, whereas Gaudreau and Blondin [14] identified three higher order dimensions (task, distraction and disengagement-oriented coping).

The use of any particular coping strategy does not guarantee its effectiveness; rather, its effectiveness depends on the context in which it is employed [7]. A number of approaches to coping effectiveness have been put forward [15]. The goodness-of-fit approach to coping effectiveness [16] has found that problem-focused coping strategies are more adaptive to athletic performance than either emotion-focused or avoidance coping strategies [3]. However, some avoidance coping strategies can be effective in dealing with acute stressors. Blocking (i.e., shut out thoughts, mentally withdraw from stressor) has been found to be an effective coping strategy in elite rugby union athletes [17]. The latter provides support for the choice of coping strategy [18] to coping effectiveness.

Personality has been found to influence the stress-coping process directly by restricting or assisting the use of specific coping strategies and indirectly, by influencing the type and intensity of the stressors experienced or coping effectiveness [19]. There is also evidence that personality has an influence on athletic performance. For example, Piedmont, Hill, and Blanco [20] showed that 23% of variance of coaches rating of female soccer players was predicted by their neuroticism and conscientiousness. Also, conscientiousness explained 8% of the variance in game statistics. Crust and Clough [21] showed that higher levels of mental toughness were associated with better performance on a physical endurance task. However, to our knowledge no study has examined how personality influences coping during actual sporting performance. A personality type which has received significant attention in the health domain is the distressed or Type D personality. Individuals with Type D personality experience a variety of negative emotions (NA; negative affectivity) and exhibit an inability to express emotions and/or behaviors in social interactions (SI; social inhibition) [6]. It is the interaction or synergetic effects of NA and SI which characterizes the Type D personality. Individuals high on NA are more likely to experience across time and situation negative emotions, depressed mood, anxiety, hostility, irritability, a negative self-view and an attentional bias towards adverse stimuli. Individuals high on SI feel inhibited, tense, have fewer personal ties, and feel uncomfortable and insecure in encounters with other people [22].

Type D personality represents a relatively homogeneous subgroup of personality traits grounded in psychological theory [23]. The subcomponents NA and SI are well represented within the five-factor framework of personality (supporting its construct validity) sharing less than 50% of the variance with neuroticism and extraversion [6]. Type D personality has incremental value above and beyond the five factor trait model [24] which suggests that Type D personality is a distinct construct. Individuals with Type D personality (those who score > 10 on both NA and SI on the DS14) appear to experience higher levels of chronic stress, emotional and social difficulties and adverse health events. Type D personality is closely associated with symptoms of depression and anxiety, chronic tension, pessimism, lack of perceived social support, lower subjective well-being and self-esteem, dissatisfaction with life, lower quality of life, and poor body image. It is also a predictor of adverse clinical events and poor coronary heart disease prognosis [22, 25–27].

In non-clinical populations, Type D personality is related to major psychosocial stressors [28], greater cardiovascular and neuroendocrine reactivity [29], increased cardiac output in response to acute laboratory stressors [30], and a hemodynamic maladaptation to an acute mental arithmetic stressor in women [31]. Type D individuals are also more likely to appraise stressful events as a threat and higher levels of perceived stress [32] and use more passive, maladaptive avoidance coping strategies, which are associated with burnout, decreased self-reported health and morale [33–35]. There is some evidence suggesting that when experiencing an acute laboratory stressor Type D individuals show greater stress reactivity [29]. However, studies have not yet examined the effects on actual (sport) performance.

Type D personality has been shown to be associated with detrimental health behaviors (e.g., more smoking) and avoidance of facilitative health behaviors (e.g., physical activity), adverse health outcomes as well as poor coping with stress [36–38]. However, to date no study has examined how Type D personality manifest itself in the realm of sport. This is an important research endeavor since sport participation has the potential to facilitate emotional regulation, which is an important need for individuals with Type D personality. Hence, being physically active has been associated with improved affective states [39]. In addition, understanding the relationship between Type D personality and stress and coping in sport can help inform effective applied stress management interventions. As such, the aim of study one was to examine the relationship between Type D personality, stress intensity appraisal of a self-selected stressor, coping, and perceived coping effectiveness in a sport context. We predicted that athletes classified as Type D would perceive the self-selected sport stressors with higher levels of intensity and would mainly use avoidance coping and emotion-focused strategies, and report lower levels of problem-focused coping. No a priori prediction was made with regard to coping effectiveness. The aim of study two was to examine the role of Type D on performance on a novel sport task as well as pre-performance anxiety levels, coping, and post-performance affect. For study two we predicted that Type D personality would be associated with increased levels of anxiety, lower levels of self-confidence prior to completing a rugby league novel task, poorer performance, and more negative emotions post-performance.

## Study1

### Materials and methods

#### Participants

Participants were 482 British, mainly Caucasian (93.8%) athletes (male *n* = 305; female *n* = 177) aged between 16 to 45 years (*M* age = 20.44 years, *SD* = 3.98), with experience in their sport from 1 to 35 years (*M* = 9.63, *SD* = 4.69). Athletes were recruited from different Universities (68%) and sports clubs in the region of Yorkshire, UK. See also Table 1 for additional demographic information. The study was approved by a University’s Research Ethics Committee and participants provided written informed consent prior to participating.

**Table 1:** Sample characteristics of the athlete participants in study 1 and difference between Type D and non-Type D athletes.

Variable	N	Total Sample	N	Non-Type D	N	Type D	*p*
Age, years (M, SD)	482	20.4 (4.0)	342	20.4 (4.1)	140	20.5 (3.6)	.81
Males (N, %)		305 (63.3)		221 (64.6)		84 (60.0)	.35
Years in sport (M, SD)	482	9.6 (4.7)	342	9.9 (4.8)	140	8.9 (4.6)	.02
Skill level (N, %)	482		342		140		.007
No response		12 (2.5)		7 (2.0)		5 (3.6)	
University/club		175 (36.3)		110 (32.2)		65 (46.4)	
County		220 (45.6)		174 (50.9)		46 (32.9)	
National		60 (12.4)		40 (11.7)		20 (14.3)	
International		15 (3.1)		11 (3.2)		4 (2.9)	
Classification (N, %)	481		341		140		.19
Contact		267 (55.4)		196 (57.5)		71 (50.7)	
Non-contact		214 (44.5)		145 (42.5)		69 (49.3)	
Pursuit (N, %)	481		341		140		.20
Team		323 (67.0)		235 (68.9)		88 (62.9)	
Individuals		158 (33.0)		106 (31.1)		52 (37.1)	
Stress Intensity	342	6.2 (2.3)	342	6.0 (2.4)	140	6.7 (2.1)	.002

#### Instruments

First, the participants completed a demographic section. This included questions on their gender, age, sport (i.e., team/individual; contact/non-contact), years they participated in their sport, skill level (i.e., University/club, county, national or international).

The Type D scale 14 (DS14) provides a taxonomic and continuous assessments of distressed personality by measuring the traits of negative affectivity (NA, 7-items; e.g., "I often feel unhappy") and social inhibition (SI, 7-items; e.g., "I am a closed kind of person"). Participants respond using a 5-point Likert scale anchored at 0 = false to 4 = true. Based on cluster analysis and item response theory, a score of 10 or more on both subscales indicates the likelihood of a respondent fitting the Type D personality profile [40]. In addition to its taxonomic use the NA and SI subscales can be multi-plicatively combined to produce a continuous measure of distressed personality, with higher scores corresponding to a greater likelihood that the respondent behaves in a manner consistent with the distressed personality type. This dimensional interpretation of distressed personality is supported by psychometric evaluations of the DS14, which have indicated that item responses are additive at the subscale and total scale levels [41, 42]. The DS14 total score (α = .88) and subscale scores (NA, α = .86; SI, α = .80) were internally consistent in the present sample, in accordance with previous studies [6].

The Modified Cope Inventory (MCOPE) [43] has 12 coping scales each consisting of four items which can be classified under 3 higher order dimensions (1) Problem focused coping: Active coping, seeking informational social support, planning, suppression of competing activities, increasing effort; (2) Emotion focused coping: Seeking emotional social support, humor, venting emotions, self-blame, wishful thinking; (3) Avoidance coping: Behavioral disengagement, denial. There is extensive evidence supporting the convergent validity of the MCOPE subscales and the MCOPE subscales have demonstrated acceptable levels of internal consistency in previous research [43, 44]. To measure coping effectiveness a 5-point Likert scale was added to the MCOPE which was anchored by 1 = extremely ineffective and 5 = extremely effective [17]. The three higher order dimensions achieved acceptable levels of reliability (Cronbach alpha between .76 and .89 for coping and coping effectiveness).

Prior to completing the MCOPE, participants reported the most intense sport stressor experienced in the last 14 days and appraised the stressor in terms of how much stress the event caused by dissecting a 10 cm bipolar line anchored by ‘not at all stressful’ vs. ‘extremely stressful’. The ‘stress thermometer’ has already demonstrated normal distribution properties and adequate variability for male and female athletes [45]. The MCOPE was completed in relation to the self-reported stressor.

#### Procedure

Athletes and coaches of sports teams received letters detailing the nature of the study and participant requirements. If the coaches granted permission for the data collection, an information letter and consent form was distributed to athletes. Research assistants, who had received training in quantitative data collection techniques, administered the paper based questionnaire pack in the same order prior to or following training sessions. All of the participants were actively involved in competitive sport and had participated competitively within 14 days of the questionnaires being administered.

#### Analysis strategy

The statistical analysis was conducted with SPSS version 22. After screening for outliers and normality, Cronbach alphas and descriptive statistics for all study variables were obtained. The influence of Type D personality on stress and coping was examined using both the taxonomic and continuous approach. For the taxonomic approach we first conducted a test to examine differences in stressor intensity as a function of Type D personality. Following this we conducted multivariate analysis of variance (MANOVA) to test differences in coping and coping effectiveness at the dimensional level. Type D was entered as a fixed factor for each analysis, and the coping dimensions were entered as the dependent variable in each analysis. MANOVA main effects were further analyzed with Bonferonni adjusted univariate analysis of variance (ANOVA) to test between group differences (Type D vs. non-Type D athletes).

We conducted regression analysis to examine the role of Type D personality as a continuous variable. Stress, and coping and coping effectiveness at the dimensional level were the dependent variables and NA, SI entered at step one and centered NA X SI at step two, the predictor variables.

### Results study 1

The coping dimensions were multivariate normal when regressed on Type D. Box’s M and Levene’s test were not significant for the variables. Table 1 shows the distribution of Type D personality among the athlete participants in the present study based on demographic variables. Type D athletes reported fewer years of participation in sport than non Type D athletes (t (480) = -2.17, P = .03; *d* = -0.20) and were comparatively less likely to compete at a regional or county level and more likely to compete at a University or local level (χ^2^ (4, 482) = 14.14, P = .02; Phi = .17). Type D groups were comparable with respect to age, gender, sport classification (contact vs. non-contact) and pursuit (team vs. individual sport).

#### Taxonomic analysis

The t-test for stressor intensity was significant (t = -3.07, P = .002; Cohen *d* = 0.32) with a small effect size and Type D individuals reporting higher levels of stress intensity.

Table 2 shows the mean standard deviations, and results, for coping and coping effectiveness at the dimensional level for the Type D and non-Type D athletes. The MANOVA for coping (Wilks’ λ = 0.94; *p* < .001, η2p = .06) and coping effectiveness (Wilks’ λ = 0.94; *p* < .001, η^2^p = .06) were both significant with small effect sizes. As predicted, Type D athletes reported less frequent use of problem- and emotion-focused coping but more avoidance coping. Type D athletes also reported lower coping effectiveness for problem-focused coping but higher coping effectiveness for avoidance coping.

**Table 2:** Mean and standard deviations for each of the coping dimensions and coping effectiveness (CE) for the Type D and non-Type D participants and results of the analysis of variance (significant results only).

	Non-Type D		Type D		Results Coping			Results CE		
	Coping	CE	Coping	CE	F(1,480)	*p*	Eta	F(1,480)	*p*	Eta
Problem Focused Coping*^$^	3.30 (.50)	3.02 (.44)	3.16 (.55)	2.83 (.46)	7.38	.02	.02	16.40	< .001	.03
Emotion Focused Coping*	2.55 (.64)	2.41 (.51)	2.73 (.69)	2.43 (.55)	6.97	.009	.02		n.s	
Avoidance Coping*^$^	1.85 (.63)	2.13 (.79)	2.10 (.73)	2.28 (.74)	13.46	< .001	.03	4.00	.046	.01

* Denotes significant difference for coping between the Type D and non-Type D group.

^$^ Denotes significant difference for coping effectiveness between the Type D and non-Type D group.

#### Continuous analysis

The regression analysis for stress intensity was significant at step one (F (2,479) = 11.51; P < .001) but not step two with NA being a significant predictor (Beta = .199; P < .001). Table 3 provides the results of the regression analysis for Type D personality as a continuous variable. As predicted, for emotion-focused and avoidance coping the interaction between NA and SI added small but significant variance to the model. However, no such effect was found in problem-focused coping.

**Table 3:** Results of the regression analysis for Type D as a continuous variable (continuous analysis).

	Coping		Coping Effectiveness
	Step 1 R^2^ and Beta predictors	Step 2 ΔR^2^	Step 1 R^2^ and Beta predictors
Problem Focused Coping	5.7%**; NA = -.141**; SI = -.131**	n.s.	5.7%**; NA = -.141**; SI = -.131*
Emotion Focused Coping	6.4%**; NA = .294**; SI = .122*	1.4%**; NAxSI = .389**	n.s.
Avoidance Coping	7.8%**; NA = .301**	1%*; NAxSI = -.328*	n.s.

*P < .05

**P < .01

NA = Negative affectivity; SI = Social inhibition

## Discussion study 1

As predicted, the results of study one suggest that Type D personality influences the stress-coping process in sport. Athletes with Type D reported higher levels of stress independent of stressor type and made use of more emotion-focused and avoidance coping strategies and less use of problem-focused coping strategies. In addition, Type D athletes reported the emotion-focused strategies as more effective, and the problem-focused coping strategies as less effective.

As predicted, and supporting previous work in organizational settings [46] and with university students [33], athletes classified as Type D reported higher levels of stress intensity independent of stressor type. The continuous analysis suggests this was mainly due to higher levels of NA. These results are not dissimilar to a study by Schoormans, Husson, Denollet and Mols [47] in a sample of cancer survivors and provide some support for the notion that competing in sport is more stressful for Type D than non-Type D athletes. On the other hand, sport participation can facilitate emotional regulation of individuals with Type D. Regular and acute bouts of exercise have been shown to have positive effects on affective states [39]. It might be that sport participation moderates the experience of stress, making the differences between Type D and non-Type D individuals less pronounced in such environments. This issue would warrant further investigation because it provides a potential mechanism for intervention to help individuals with Type D to reduce their stress levels.

Both the taxonomic and continuous analysis supported the predictions that Type D was directly associated with significantly more use of emotion-focused and avoidance coping whereas the taxonomic analysis also indicated less use of problem-focused coping [33, 35]. The finding that Type D is associated with more use of emotion-focused coping might be due to the fact that participants experienced more intense emotions because of higher levels of stress. This is supported by recent findings that Type D is associated with lower HRV which in turn is associated with greater social threat and anxiety [48]. A priority for Type D athletes would be to down regulate such emotions. Lower resting HRV as found by Jandackova et al. [48] in Type D individuals is associated with reduced context appropriate emotional responses [49]. However, there was no difference in coping effectiveness for emotion-focused coping suggesting that Type D athletes might have developed strategies to deal with increased emotionality and stress. The physiological and psychological responses to being physically active might influence the experience of stress and emotions as well as the coping responses. However, this would require further study.

Avoidance coping is likely to be used in situations in which the athlete experiences limited power to change the outcome of a situation [50]. Such coping strategies can be adaptive if the athlete is unable to achieve their goal regardless of investment of effort [51, 52]. Avoidance coping is not an uncommon strategy used by athletes. Nicholls, Jones, Polman, and Borkoles [53] for example found that professional rugby union athletes used blocking as one of the most frequent coping strategies during training and competition. Although providing temporary relief from an acute stressor, in the long-term avoidance coping is maladaptive and can result in adverse psychological and physiological outcomes including higher stress levels [50, 54].

Most studies report that athletes make a greater use of problem-focused coping in comparison to emotion-focused or avoidance coping [1]. Problem-focused coping involves active problem solving and is regarded more adaptive in nature. For example, problem-focused coping is associated with positive affect [55] and subjective performance ratings [3]. The taxonomic analysis showed that individuals classified as Type D used less problem-focused coping and rated this form of coping as less effective.

The present findings support the cognitive-motivational-relational model of stress and coping [7], which predicts that variables, including personality, influence how frequently coping strategies are used and how effective they are perceived to be. The study's observational design precludes inferences about the causal links between personality, stress and coping. For example, it is possible that stressor intensity confounds the association between Type D personality and coping. However, the pattern of results may prove informative for future research.

The present results are also consistent with a functional account of behavior [56, 57]. In particular, the tendency of Type D individuals to perceive stressors as more intense in conjunction with their inclination to use avoidance and emotion-focused coping, and their disinclination to use problem-focused coping. A functional viewpoint conceives of coping as behavior that is based on predictions of one's own future behavior, and that seeks to maximize long-term rewards [56].

Distressed personality implies a susceptibility to acute stress, which may engender reliance on coping strategies that have utility in situations of acute stress. The utility of avoidance strategies becomes evident if one considers that the negative affect associated with avoidance coping may serve as a substitute for emotional states that are relatively more damaging to performance and self-esteem, such as shame associated with the prospect of failure. This interpretation implies that Type D personality, through the preferential use of avoidance and emotion-focused coping, are primarily motivated to manage aversive emotional states induced by stressors. They may correspondingly be less inclined (or less able) to cope by responding to aspects of the stressor that exist apart from its emotional impact, as is characteristic of problem-focused coping. The latter coping process implies a sensitivity to long-term contingencies that reach into the past and extend into one's future or imagined prospects. By contrast, so-called maladaptive coping strategies may predominantly arise from short-term contingencies whose immediate consequences are paramount [56]. This interpretation predicts that individuals responding to acute stress will be inclined to use coping strategies that have short-term utility because such strategies ostensibly forestall aversive emotional states in the short term. However, habitual use of avoidance coping, for instance, is likely to be a strategy of diminishing returns [56, 58]. Over the long run, avoidance coping may consequently prove detrimental to athletic performance. Study two investigates the effect of Type D personality and affective states on objective measures of athletic performance of athletes.

## Study 2

### Material and methods

#### Participants

Thirty-two healthy Caucasian British male university student athletes (M age = 20.5 years; SD = 1.0 years) took part in study two. Participants reported M = 11 (SD = 4.2) years of sporting experience, played M = 4.2 (SD = 3.1) competitive games per month, trained M = 2.38 (SD = 1.12) times per week with session of M = 78.9 (SD = 33.8) minutes' duration. The effort exerted was rated M = 7.7 (SD = 1.7) on the ten point Borg scale. The main sport of 27 participants was soccer, followed by cricket (n = 1), athletics (n = 1), fitness (n = 2) and tennis (n = 1). The study was approved by a University ethics committee. In addition, all participants provided written informed consent prior to their involvement in the study.

#### Instruments

Participants first completed a demographic section consisting of questions regarding age, ethnicity, sport played, years of participation, time spent training, duration and perceived effort in training and frequency of competition. Following this they completed the DS14.

Pre-performance state anxiety and self-confidence was assessed with the Competitive State-Anxiety Inventory-2 Revised [59]. The CSAI-2R consist of 7-items measuring somatic anxiety, 5-items cognitive anxiety and 5-items self-confidence and is scored on a 4-point Likert scale (1 = not at all to 4 = very much). The CSAI-2R has been shown to have a good factorial structure and internal consistency [59]. In the present study internal consistency was good for the somatic anxiety (α = .82) and self-confidence (α = .80) scales and adequate for the cognitive anxiety scale (α = .67).

Post-performance affect was examined using the Positive and Negative Affect Scale (PANAS) [60]. The PANAS is scored on a 5-point Likert scale (1 = not at all to 5 = extremely) with 10 items measuring positive affect and 10 items measuring negative affect. Higher scores on each subscale indicate a greater presence of affect it measures. Both scales have been shown to be uncorrelated and stable over a 2 month period, with high internal consistency [60]. Internal consistency in the current study was acceptable (Cronbach’s α = .87 and .70 for PA, NA respectively). For both the CSAI-2 and PANAS the participants were asked to complete the instrument with how they felt ‘right now’.

Because of coping being assessed immediately following performance and the inclusion of other constructs this study made use of the much shorter Brief Approach/ Avoidance Coping Questionnaire (BACQ) [61] to reduce the burden on participants. The BACQ is a 12-item construct, measuring three distinct coping dimensions. Six-items assess general approach coping, 3-items cognitive avoidance coping and 3-items measure resignation/ withdrawal. The scale is anchored by a 5-point Likert scale (1 = disagree completely to 5 = agree completely). Previous research has shown the BACQ to be internally consistent [61]. Weak internal consistency was shown for the cognitive avoidance (α = .55) and resignation/ withdrawal (α = .66) scales and good reliability for the approach coping scale (α = .83). Considering the low number of scale items [62], item total correlations [63] and arguments set out in study one all subscales were used for statistical analysis.

#### Procedure

Participants were recruited from one University in England through advertising and referrals. On the first visit to the laboratory participants were informed about the nature of the study before providing consent. Following this a questionnaire pack was completed. On the second visit the participants were made aware of previous performance charts regarding distance and success on the task to be completed. They were then provided with standard instructions regarding the aims and objectives of the task before completing the CSAI-2R.

The experimental task consisted of a rugby league circuit and was new to all participants. The task was extensively piloted to test its suitability and how effectively it induced stress. The task consisted of participants running as many circuits in 3 minutes in a sports hall. From a start line, 10 m were run before returning to the line. Once turned, a further 20 m were run, again returning to the start. The individual turned and ran 30 m, before progressing 5 m to a cone station located on the left. Side-stepping around 6 cones placed 1 m apart and 0.5 m away from the center, in an alternating fashion, followed by 5 m of running. On the first passing station, 4 rugby balls were passed from left to right, at a target 8 m away, 1.50 m parallel to the floor and 0.5 m in diameter. A 5 m length ladder was located 5 m from the first passing station, requiring one foot placed in each section of the ladder. Travelling a further 5 meters, a second passing station was positioned. Four rugby balls were passed from right to left, at a target 8 meters away, 1.50 m parallel to the floor and 0.5 m in diameter. The individual ran 5 meters back to the start and repeated until 3 minutes expired. Running one circuit lasted approximately 50 seconds, and was 130 meters in distance. The outcome measures for the performance task were distance travelled in 3 minutes and the number of errors made (targets missed).

To increase anxiety participants were provided with verbal prompts during the circuit: ‘you are falling behind, what are you going to do about it (30 sec); ‘you are 5 seconds adrift, keep working (90 sec), and ‘last 30 seconds, put everything into it’ (150 sec). Also, £50 was offered to the participant who performed the best on the circuit as assessed by a Great Britain rugby league player. To this end a video camera was used to film each performance. The researcher filmed performance, with two assistants as ball collectors. Shuttle run and ladder placement made achievement of success on the passing stations more difficult. Following this the participants completed the PANAS and BACQ which was followed by a debriefing.

#### Data analysis

Descriptive statistics for all dependent variables were calculated. Independent sample t-test were conducted to examine differences between those classified having Type D personality (NA and SI > 10) and non-Type D participants. Minimum group sizes of 12 participants were required to achieve an 80% chance of detecting a large effect (Cohen’s *d* > 1) at a two-tailed α level of .05 [64]. We did not use regression analysis in this study because of lack of power due to its small sample size. Statistically significant contrasts are reported with bias-corrected (Hedges’) Cohen’s *d* effect sizes with 95% confidence intervals.

### Results study 2

Ten participants were classified as Type D and 22 as non-Type D. The two groups did not differ on the demographic variables except duration of training. Type D participants (M = 57.58) trained for a considerably smaller length of time per session than non-Type D (M = 88.64) participants (z = 2.42, P = .01).

Table 4 provides the means and standard deviations for the two groups for the dependent variables as well as the results of the statistical analysis. Pre-performance the non- Type D athletes reported significantly higher scores for self-confidence than the Type D participants with a large effect size (t30 = -3.07; P = .003; *d* = 1.20; 95% CI 0.40 to 2.00). Although the Type D participants reported higher somatic and cognitive anxiety these were not significantly different from the non-Type D participants.

**Table 4:** Mean and standard deviations and statistical result for the dependent variables of study 2.

	Non-Type D (n = 22)		Type D (n = 10)				CIs difference	
	*M* (*SD*)		*M* (*SD*)		*t30*	*p*	Lower Upper	
**CSAI**						ns		
Somatic Anxiety	17.73	5.82	20.71	6.43		ns		
Cognitive Anxiety	17.45	5.42	20.40	4.97		ns		
Self-Confidence	29.45	5.79	22.00	6.60	-3.07	.003	-12.2	-2.7
**PANAS**								
Positive Affect	32.05	6.35	35.00	4.98		ns		
Negative Affect	12.86	2.23	14.50	4.14		ns		
**BACQ**								
Approach	21.18	4.01	20.50	5.48	-	ns		
Avoidance	9.27	2.14	9.10	2.02	-	ns		
Resignation/withdrawal	5.41	2.13	7.80	2.34	2.85	.008	0.68	4.10
**Performance**								
Distance (m)	372	19.9	320	25.7	-6.23	< .001	-69	-35
Targets missed	9.5	2.5	15.6	2.7	6.24	< .001	4.1	8.1

During performance of the 3 minute rugby league circuit task the Type D participants covered significantly less distance (t(30) = -6.23; P < .001; *d* = -3.04; 95% CI -4.00 to -1.99) and made more errors (t(30) = 6.24; P < .001; *d* = 2.38; 95% CI 1.38 to 3.26) than the non- Type D participants. The effect sizes were large for both distance travelled and errors made.

Post-performance the Type D and non-Type D participants did not report any differences in either positive or negative affect. However, Type D participants reported more use of resignation/withdrawal coping (t(30) = 2.85; P = .008; *d* = 1.06; 95% CI 0.27 to 1.85) but not approach or avoidance coping strategies.

### Discussion study 2

Type D individuals in study two reported lower levels of self-confidence prior to completing the rugby league circuit task. In addition, they performed poorer as indexed by less distance completed and more mistakes made. This was associated with more frequent use of resignation/withdrawal coping. No differences in post-performance affect were observed.

Previous studies have indicated that Type D individuals report increased levels of stress and show higher levels of stress reactivity in laboratory tasks [30, 31]. This is the first study to indicate that Type D personality is also associated with decreased task performance. In addition, poorer performance was associated with lower self-confidence prior to the task and increased use of resignation/withdrawal coping after completion of the task. This is supported by findings that Type D individuals have a tendency to search the environment for trouble [65] and report lower levels of general self-efficacy [37]. The lower levels of self-efficacy in the study by Wiencierz and Williams [37] were associated with significant less total exercise participation and walking in Type D versus non Type D individuals. Our findings also support meta-analytic findings in sport [66] which have shown that self-confidence in particular (r = .24) has an influence on performance whereas cognitive anxiety (r = -.10) has a minimal influence.

Few studies have examined the association between coping and objective measures of performance. Our results suggest that the increased use of resignation/withdrawal coping by the Type D participants is associated with poorer performance [3]. We deliberately used a shorter coping questionnaire for this study to reduce demands on the participants. However, in line with study one it appears that Type D is associated with the use of more maladaptive coping strategies. More importantly, this is the first study to demonstrate that Type D personality negatively influences actual performance in sport rather than self-rated performance satisfaction [3].

No difference was found in post-performance affect between the Type D and non- Type D participants. This finding provides partial support for the observation from study one in that sport might provide an outlet for Type D individuals to regulate their emotions. This could be partly due to the fact that exercising, albeit at moderate intensity, is associated with positive affect [39]. An important limitation of the current study is the generalizability of the rugby task to real sporting competitions. Although the task selected has many similarities to real rugby competition behavior the environment in which it was conducted was very different. The task however allowed for the quantification of completion time and error. Future research could identify and examine behaviors which reflect this in real sporting competitions.

## Overall discussion

This is the first study to examine the role of Type D personality, stress, coping, and performance in the context of sport. There is ample evidence that Type D personality has a negative impact on mortality and morbidity in CHD patients [26] and the general population [25]. The present study indicates that Type D personality has consequences for dealing with stress during competition and negatively affects performance in sport.

From a theoretical perspective, this study adds to our understanding of factors which influence the stress and coping process as well as athletic performance. The findings indicate that Type D personality influences actual sport performance and that the use of coping strategies that are maladaptive in the long run by Type D athletes may explain this finding.

In sport [67], health and medicine [68] individual or personalized approaches have been advocated. Although Type D is characterized by high levels of NA and SI the psychosocial, health and behavioral consequences are likely to vary across populations and settings. Based on our findings interventions for Type D athletes could focus on mood modification and enhancing interpersonal functioning [69]. With regard to mood modification it has been shown that a mindfulness-based stress reduction training program reduced NA and SI in the general population. Increased proficiency in mindfulness was found to be the main mechanism for this reduction [70]. Also, research in the sport context [71] suggests that higher levels of mindfulness are associated with reduced stress and improved coping effectiveness. Hence, future investigation are recommended to test the usefulness of mindfulness practice among Type D athletes. In addition, relaxation training (e.g., autogenic training, progressive muscle relaxation, relaxation imagery) or psychosocial coping interventions might be beneficial to Type D athletes. Psychosocial interventions could in particular target the appraisal process (perceive a stressful event as a challenge rather than a threat) through cognitive restructuring, development of emotion-focused skills to down regulate their emotional state (e.g., breathing), developing their problem-focused coping repertoire (e.g., planning, increasing effort) whilst reducing maladaptive avoidance coping strategies. Interpersonal functioning of Type D individuals could be improved through assertiveness training. This would allow Type D athletes to use more active and adaptive coping strategies in stressful encounters rather than using passive maladaptive coping strategies.

Our studies are not without limitations. Study one had a cross-sectional design which cannot support causal inferences. Also, athletes self-reported retrospectively on the stress experienced and coping used to deal with a stressful event. This can result in bias and forgetfulness. The sample used in study one was also heterogeneous in nature. In study two only males participated, as such it is unclear whether findings can be generalized to females. In addition, future research has to establish whether the task selected generalizes to behaviors in actual sporting competitions.

In conclusion, our novel findings suggest that Type D personality is associated with the use coping strategies which are considered to be maladaptive in the context of sport. In addition, Type D personality is associated with poorer performance on a sporting task which was associated with lower levels of self-confidence and more use of resignation/withdrawal coping.

## Supporting information

S1 Data set This is the dataset for study 1. (SAV)

S2 Data set This is the dataset for study 2. (SAV)
